# Caspase-mediated proteolysis of the sorting nexin 2 disrupts retromer assembly and potentiates Met/hepatocyte growth factor receptor signaling

**DOI:** 10.1038/cddiscovery.2016.100

**Published:** 2017-01-23

**Authors:** Catherine M Duclos, Audrey Champagne, Julie C Carrier, Caroline Saucier, Christine L Lavoie, Jean-Bernard Denault

**Affiliations:** 1 Department of Pharmacology-Physiology and Institut de Pharmacologie de Sherbrooke, Faculty of Medicine and Health Sciences, Université de Sherbrooke, 3001, 12th Avenue North, Sherbrooke, QC, Canada J1H 5N4; 2 Department of Anatomy and Cell Biology, Faculty of Medicine and Health Sciences, Université de Sherbrooke, 3001, 12th Avenue North, Sherbrooke, QC, Canada J1H 5N4

## Abstract

The unfolding of apoptosis involves the cleavage of hundreds of proteins by the caspase family of cysteinyl peptidases. Among those substrates are proteins involved in intracellular vesicle trafficking with a net outcome of shutting down the crucial processes governing protein transport to organelles and to the plasma membrane. However, because of the intertwining of receptor trafficking and signaling, cleavage of specific proteins may lead to unintended consequences. Here we show that in apoptosis, sorting nexin 1 and 2 (SNX1 and SNX2), two proteins involved in endosomal sorting, are cleaved by initiator caspases and also by executioner caspase-6 in the case of SNX2. Moreover, SNX1 is cleaved at multiple sites, including following glutamate residues. Cleavage of SNX2 results in a loss of association with the endosome-to-*trans*-Golgi network transport protein Vps35 and in a delocalization from endosomes of its associated partner Vps26. We also demonstrate that SNX2 depletion causes an increase in hepatocyte growth factor receptor tyrosine phosphorylation and Erk1/2 signaling in cells. Finally, we show that SNX2 mRNA and protein levels are decreased in colorectal carcinoma and that lower *SNX2* gene expression correlates with an increase in cancer patient mortality. Our study reveals the importance to characterize the cleavage fragments produced by caspases of specific death substrates given their potential implication in the mechanism of regulation of physiological (signaling/trafficking) pathways or in the dysfunction leading to pathogenesis.

## Introduction

Apoptosis is a mechanism conserved in multicellular organisms that is crucial for the elimination of unwanted or damaged cells, normal development, cell homeostasis and the regulation of the immune system. This cell death process depends on the activation of the caspase family of peptidases and their ability to proteolyze specific substrates following aspartate residues (clan CD, family C14),^1–4^ although a recent study showed that caspases can also cleave following glutamate residues.^5^ Apoptotic caspases are classified into two main categories: the initiators (caspases 2, 8, 9 and 10) and the executioners (caspases 3, 6 and 7).

During apoptosis, caspases cleave hundreds of proteins belonging to diverse functional protein groups such as cytoskeletal and structural proteins, DNA replication and repair, signal transduction and intracellular trafficking.^6,7^ Proteins from the latter group are generally inactivated to interrupt protein sorting, transport and delivery to organelles, and within the secretory and endocytic pathways. For instance, cleavage of the small G-protein Rab5 effector rabaptin-5 during apoptosis results in the loss of endosome fusion and halts the endocytic pathway,^8,9^ whereas caspase-mediated proteolysis of syntaxin 5 and giantin, two Golgi-localized proteins involved in vesicular transport, results in a block of the trafficking between the ER and the Golgi.^10^

According to proteomic studies, the two sorting nexins (SNX1 and SNX2) are cleaved during apoptosis.^7,11,12^ SNXs belong to a 33-member family involved in various functions in protein trafficking.^13^ SNX1 and SNX2, which are part of the SNX-BAR (Bin/Amphiphysin/Rvs) subfamily, are ubiquitous proteins sharing 63% residue identity. They both possess an unstructured N-terminal domain (NTD) thought to mediate protein–protein interactions. The NTD is followed by the phox homology (PX) domain that preferentially recognizes phosphatidylinositol-3-phosphate-enriched endosomal membranes and a C-terminal BAR domain responsible for their heterodimerization with other SNXs.^14–16^ The BAR domain senses the curvature of the membrane, promotes membrane tubulation and contributes with the PX domain to early endosome localization.^17,18^

An important role of SNX1 and SNX2 is to interact with the retromer, a protein subcomplex composed of vacuolar protein sorting Vps26, Vps29 and Vps35.^19,20^ This subcomplex is essential for the transport of receptors, such as the cation-independent mannose-6-phosphate receptor (ci-MPR), from endosomes to the *trans*-Golgi network (TGN) and to prevent them from reaching the lysosomes. Furthermore, depletion of SNX1 and SNX2 prevents the association of the retromer subcomplex to the endosomes and impairs ci-MPR recycling.^20^ However, it is important to note that the role of SNXs in directing ci-MPR fate is still debated as groups have observed effects that vary greatly between experimental conditions. Thus, we are far from fully understanding how SNXs work with the retromer.^21–23^ Irrespective of their association with the retromer, both SNX1 and SNX2 can interact with membrane receptors and modulate their recycling and degradation, including that of many G-protein-coupled receptors^24–28^ and receptor tyrosine kinases (RTKs), such as the hepatocyte growth factor (HGF)/Met receptor.^14,29–33^ Specifically, SNX2 interacts with Met and its depletion in lung cancer cells overexpressing constitutively active Met promotes its lysosomal degradation.^34^

In this study, we validate SNX1 and SNX2 as novel caspase substrates. We demonstrate that their cleavage is performed by initiator caspases and executioner caspase-6 *in vitro* and *in cellulo*. We validated the previously reported cleavage sites in SNX1 and SNX2, and identify 15 new cleavage sites in SNX1, including following glutamate residues. We also showed that truncated SNX2 no longer interacts with the retromer component Vps35 and provokes Vps26 delocalization. Finally, we show that depletion of SNX2 potentiates Met phosphorylation and signaling and that the expression of SNX2 is reduced in human colorectal carcinoma (CRC).

## Results

### SNX1 and SNX2 are cleaved by caspases during apoptosis

**Figure 1:**
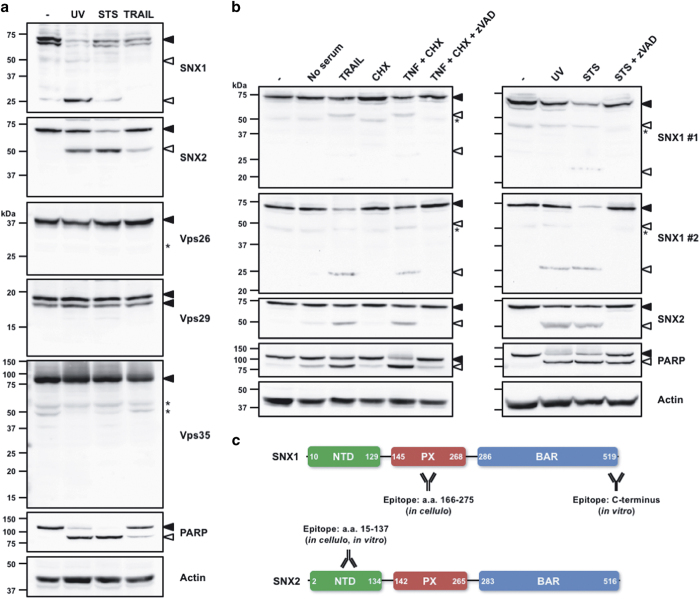
The retromer-associated proteins SNX1 and SNX2 are cleaved during apoptosis. (**a** and **b**) HeLa cells were left untreated (−) or treated for 16 h with the following conditions: UV (254 nm wavelength, 100 J/m^2^); STS (0.5 *μ*M); TRAIL (100 ng/ml) in serum-free medium; cycloheximide (CHX, 10 *μ*g/ml); tumor necrosis factor*-α* (TNF*-α*) (50 ng/ml); and Z-VAD-fmk (10 *μ*M). Proteins were analyzed by immunoblotting using the indicated antibodies. In (**b**), two different immunoblotting protocols were used to reveal cleaved SNX1 fragments. (**c**) Schematic representations of SNX1 and SNX2 proteins. The limits of domains are indicated by numbers; the epitopes recognized by the antibodies used in this study are indicated. Closed and open arrowheads indicate full-length and cleaved fragments, respectively. Actin was used as a loading control. Asterisks indicate nonspecific proteins recognized by the antibodies.

Proteomic studies have listed SNX1 and SNX2 as potential caspase substrates.^7,11,12^ Thus, their proteolysis in HeLa cells treated with various well-established apoptotic stimuli of the intrinsic (ultraviolet radiation (UV) and staurosporine (STS)) and of the extrinsic (TRAIL) apoptotic pathways was analyzed (Figure 1a). In these conditions, both SNX1 and SNX2, but not the retromer components Vps26, Vps29 and Vps35, were cleaved. Further analyses using two different immunoblotting protocols revealed that whereas SNX1 was proteolyzed into a 25- and a 50-kDa fragments, a single 50-kDa cleavage moiety was detected for SNX2 (Figure 1b). It is noteworthy that the extent of SNX1 and SNX2 cleavage mirrors that observed for the caspase-3/7 hallmark death substrate poly(ADP-ribose) polymerase 1 (PARP).^35–37^ Importantly, these proteolytic events were readily blocked by the pancaspase inhibitor Z-VAD-fmk. These results demonstrate that SNX1 and SNX2 are cleaved during apoptosis.

### Multiple caspases cleave SNX1 and SNX2 *in vitro*

**Figure 2:**
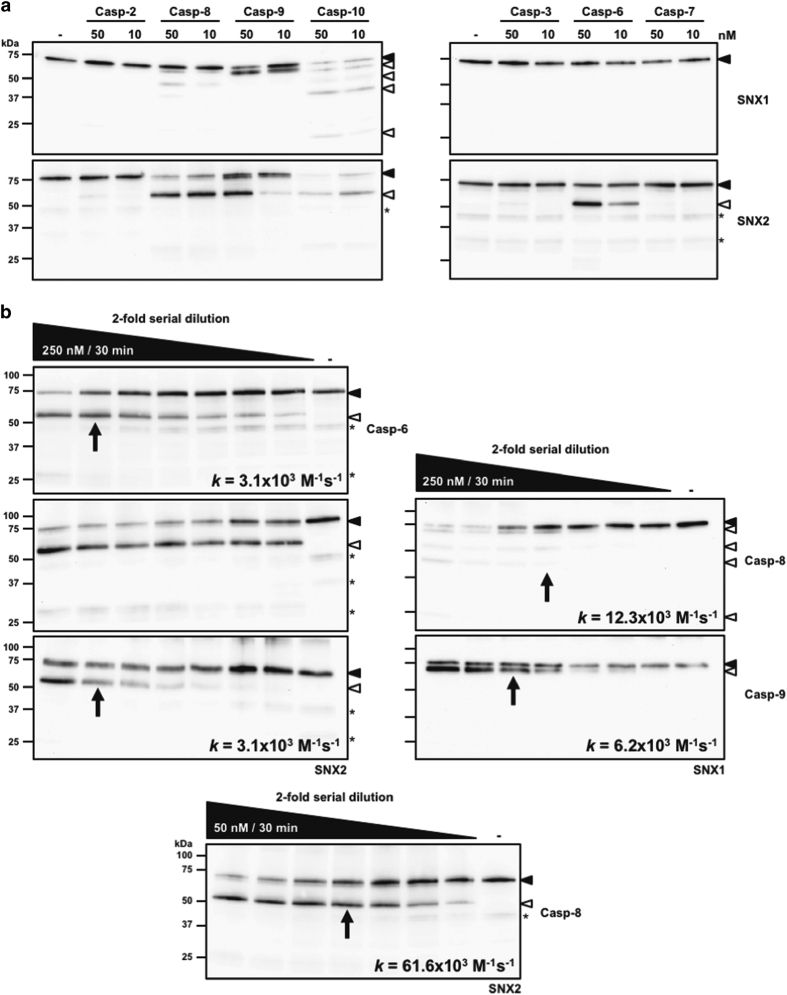
SNX1 and SNX2 are cleaved by recombinant caspases. (**a**) Cleavage assays were performed using 25 nM recombinant SNX1 or SNX2 protein and physiological concentrations (10 or 50 nM) of the indicated initiator (left panels) or executioner (right panels) caspases for an hour at 37 °C in the appropriate buffer as described in the Materials and Methods section. (**b**) Similar assays as in (**a**) were performed using twofold serial dilutions of the indicated recombinant caspases. The highest concentration of enzyme used and assay time are indicated in the black wedges. Proteins were analyzed by immunoblotting using the indicated antibodies. The rate *k* of full-length SNX disappearance was obtained from the equation *k*=ln 2/*tE*, in which *t* is time and *E* is caspase concentration at 50% protein cleavage (indicated by an arrow on each blot).^39^ Because of the high efficacy of caspase-8 at cleaving SNX2, a different range of concentrations was used (bottom panel) to estimate the cleavage rate *k*. Closed and open arrowheads indicate full-length and cleaved fragments, respectively. Asterisks indicate nonspecific proteins recognized by SNX2 antibody.

To determine which caspases are responsible for SNX1 and SNX2 proteolysis, *in vitro* cleavage assays using active site-titrated recombinant peptidases and substrates were conducted. Recombinant SNXs were incubated with physiological concentrations of caspases in the appropriate caspase assay buffer.^38^ In these optimized conditions, SNX1 cleavage was performed by initiator caspases 8–10, which generated several fragments (Figure 2a); caspase-10 was the most efficacious at cleaving SNX1 producing at least four fragments (25, 40, 50 and 65 kDa). Moreover, incubation with caspases 8 and 9 generated three (40, 50 and 65 kDa) and one (65 kDa) fragments, respectively, which represented a subgroup of those produced by caspase-10. Similarly, cleavage assays performed with up to 250 nM caspase-8 resulted in similar proteolytic pattern as that produced by caspase-10 (Figure 2b). Only one major proteolytic fragment of 50 kDa was produced from SNX2, which correspond to the one seen *in cellulo* (Figure 2a). Cleavage of SNX2 was performed by initiator caspases 8–10, but most efficaciously by caspases 8 and 10. Interestingly, caspase-6 was the sole executioner able to cleave SNX2.

Kinetic analyses using caspases 6, 8 and 9 were also performed to estimate cleavage rates (Figure 2b).^39^ Based on these assays, caspase-8 is best at cleaving SNX2, whereas caspase-9 is twice as effective as caspase-8 at cleaving SNX1. These analyses show that the 50- and 65-kDa SNX1 fragments were produced first, followed by the 40- and then the 25-kDa fragments (Figure 2b). Taken together, these results demonstrate that SNX1 and SNX2 can be cleaved by initiator caspases 8–10, and SNX2 also by caspase-6.

### Identification of caspase cleavage sites for SNX1 and SNX2

**Figure 3:**
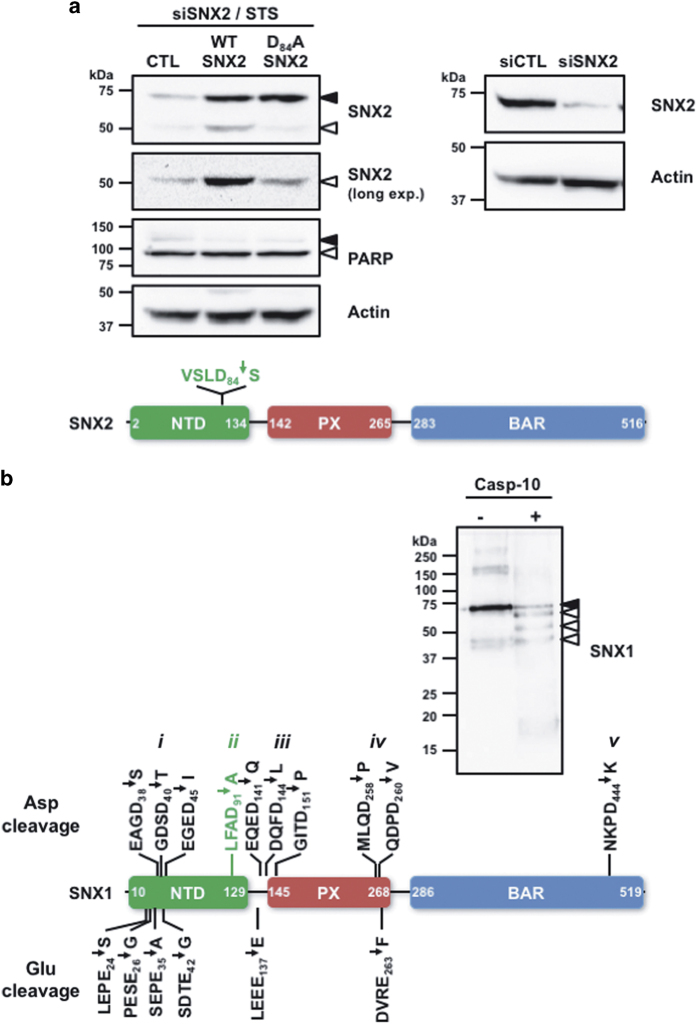
Identification of caspase cleavage sites in SNX1 and SNX2. (**a**) HeLa cells were transfected two times at 24 h interval with a SNX2 small interfering RNA (siRNA) and then with either empty (CTL), siRNA-resistant WT or Asp_84_/Ala SNX2 plasmids. Transfected cells were then incubated with STS (0.5 *μ*M) for 6 h (left panel). These conditions lead to 90.3% SNX2 depletion (right panel). The schematic presents the location of the sole cleavage site found in SNX2. (**b**) Recombinant SNX1 was incubated with or without recombinant caspase-10 for an hour at 37 °C. An aliquot of each sample was analyzed by immunoblotting using a SNX1 antibody. Samples were analyzed by tandem mass spectrometry as described in the Materials and Methods section. The schematic displays the 16 cleavage sites that were identified in SNX1; the previously identified cleavage site is indicated in green. See Supplementary Figure 1 for peptide listing and MS data used to identify these cleavage sites. Proteins were analyzed by immunoblotting using the indicated antibodies. Closed and open arrowheads indicate full-length and cleaved fragments, respectively. Actin was used as a loading control.

Cleavage of SNX2 by caspases shows a single 50-kDa fragment. Because SNX2 proteolysis following Asp_84_ is predicted to generate a 50-kDa fragment, this cleavage site was confirmed by site-directed mutagenesis. siRNA-mediated depletion of SNX2 in HeLa cells was performed and cells were transfected to rescue expression of WT or an Asp_84_/Ala mutant SNX2 (Figure 3a). STS-treated cells expressing the caspase-resistant SNX2 mutant showed cleavage level similar to that observed in control cells, demonstrating that only the remaining endogenous SNX2 was cleaved, compared with cells rescued with WT SNX2 that showed substantial cleavage.

Because several SNX1 cleavage fragments were observed, suggesting multiple cleavage sites, the identification of those sites was pursued. As caspase-10 was able to recapitulate all fragments observed with the other caspases, recombinant SNX1 was cleaved with this caspase and the reaction was analyzed by tandem mass spectrometry (Figure 3b). The results indicate that five different regions of SNX1 were subject to proteolysis, including three cleavage sites found following aspartate residues in the N terminus of the NTD and one in the C terminus of that domain, five sites in the PX domain and one site in the C-terminal end of the BAR domain. Notably, peptides revealing proteolysis following glutamate residues^5^ were also sought and six were identified, including four corresponding to cleavage in the NTD and two bordering the PX domain. Importantly, the cleavage site LFAD_91_↓A localized at the end of the NTD was also found in the analysis and this cleavage is predicted to generate a 50-kDa fragment that we observed both *in cellulo* and *in vitro*. The complete list of peptides and the corresponding mass spectrometry spectra are presented in Supplementary Figure 1. Taken together, our results validate the cleavage of SNX2 following Asp_84_ and that of SNX1 at Asp_91_ along with new caspase cleavage sites in SNX1.

### SNX2 is cleaved by caspase-6 during apoptosis

**Figure 4:**
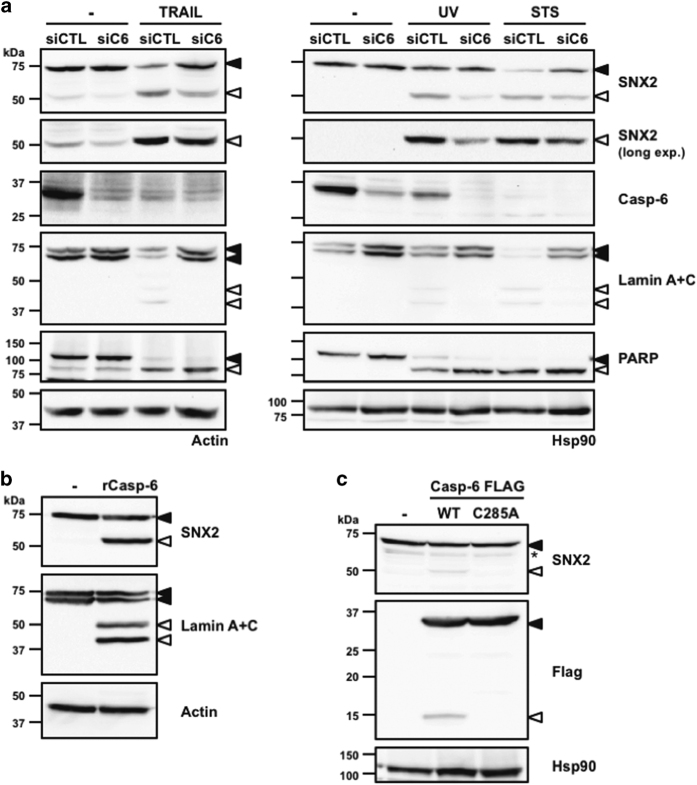
SNX2 is a caspase-6 substrate. (**a**) HeLa cells were transfected with control (siCTL) or caspase-6 small interfering RNAs (siRNAs) (siC6) followed by a 16 h treatment with TRAIL (100 ng/ml), STS (0.5 *μ*M) or UV (254 nm wavelength; 100 J/m^2^). (**b**) Eighty micrograms of HeLa cell extracts were left untreated or incubated with 250 nM active site-titrated recombinant caspase-6 for an hour at 37 °C. (**c**) HEK293T cells were transfected with either control (−), Flag-tagged WT or catalytic mutant (C285A) caspase-6 plasmids for 48 h. Proteins were analyzed by immunoblotting using the indicated antibodies. Closed and open arrowheads indicate full-length and cleaved fragments, respectively. Actin and HSP90 were used as loading controls. The asterisk indicates a nonspecific protein recognized by the SNX2 antibody.

Given that SNX2 is cleaved at a single site by caspases, we focused on this SNX to further investigate the cellular pathway leading to its cleavage. Because caspase-6 is the most downstream member of the caspase activation cascade,^40^ its depletion from cells using siRNA and the induction of apoptosis were performed (Figure 4a). Whereas caspase-6 depletion was able to abrogate the cleavage of lamins A and C, two caspase-6-specific substrates,^41^ it only partly blocked SNX2 cleavage, demonstrating that although caspase-6 participates in SNX2 proteolysis, other more upstream caspases likely contribute to its cleavage. Importantly, depleted cells showed no reduction in the proteolysis of PARP, a caspases 3 and 7 substrate. To further confirm that caspase-6 can directly cleave SNX2, HeLa cell extracts were incubated with 250 nM recombinant caspase-6, which resulted in the production of a single 50-kDa SNX2 fragment and the cleavage of lamins A and C to a similar extent (Figure 4b). SNX2 proteolysis in HEK293T cells was also detected when active caspase-6 was ectopically overexpressed (Figure 4c). These results demonstrate that SNX2 is a caspase-6 substrate.

### SNX2 cleavage disrupts its interaction with retromer components

**Figure 5:**
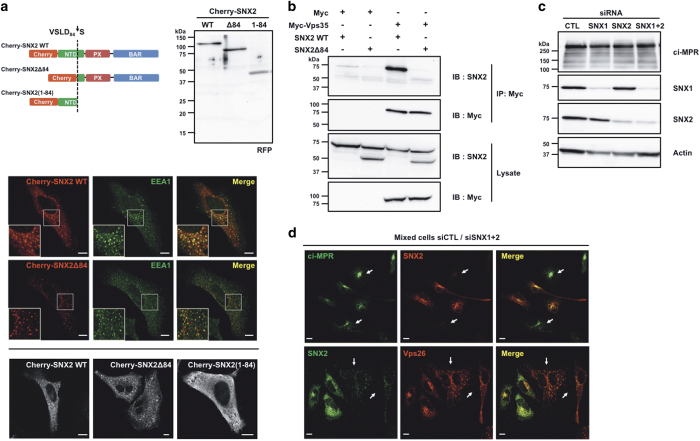
Cleavage of SNX2 disrupts interaction with Vps35. (**a**) HeLa cells were transfected for 16 h with plasmids encoding Cherry-fused full-length SNX2 (WT), C-terminal fragment (Δ84) or N-terminal fragment (1–84). Cherry-fused protein integrity was analyzed by immunoblotting using an RFP antibody (top). Colocalization with early endosomes was analyzed by confocal microscopy using SNX2, EEA1 early endosome marker and RFP antibodies (bottom). (**b**) HEK293T cells were transfected for 48 h with the indicated combination of empty (Myc), Myc-tagged Vps35, SNX2 WT and SNX2Δ84. Samples were immunoprecipitated (IP) using a Myc antibody and proteins were analyzed by immunoblotting (IB) with the indicated antibodies. (**c**) HeLa cells were transfected two times at a 24 h interval with the indicated combination of control (CTL), SNX1 and SNX2 small interfering RNAs (siRNAs), and proteins were analyzed after 72 h by immunoblotting using the indicated antibodies. Actin was used as a loading control. (**d**) Mixed populations of cells transfected as in (**c**) were analyzed for ci-MPR, SNX2 and Vps26 localization by confocal microscopy. Arrows point to SNX1/SNX2-depleted cells. Scale bars in (**a**) and (**d**) represent 10 *μ*m.

Because SNX2 is mainly known as a retromer-associated protein involved in endosome-to-TGN retrograde transport, the effect of its cleavage by caspases was analyzed first for this role. By analogy to SNX1, the NTD of SNX2 should interact with the retromer components^31^ and cleavage within this domain should disrupt this interaction. First, transfection of SNX2 mutants that mimic the cleavage products fused to the Cherry fluorescent protein was analyzed by confocal microscopy (Figure 5a). As expected, Cherry-SNX2 and Cherry-SNX2Δ84, mimicking the C-terminal fragment, localized predominantly to early endosomes, as shown by the colocalization with the early endosome antigen 1 (EEA1) marker (Figure 5a). However, Cherry-SNX2(1–84) that mimics the N-terminal fragment localized exclusively in the cytosol (Figure 5a). Second, SNX2 association with the core retromer component Vps35 was examined by co-immunoprecipitation (Figure 5b). In these conditions, WT SNX2 co-immunoprecipitated with Vps35, while SNX2 lacking the N-terminal segment failed to interact. These results show that cleavage of SNX2 decouples the retromer cargo recognition machinery from the SNXs trafficking machinery.

To investigate whether the cleavage of SNX2 alters the trafficking of cargo proteins, we next planned to analyze the levels and distribution of ci-MPR in depleted cells rescued with SNX2Δ84 and SNX2(1–84). However, whereas previous work has shown that depletion of SNX1 and SNX2 impeded the retrograde transport of the ci-MPR and promoted its lysosomal degradation,^20^ no substantial reduction of ci-MPR levels was observed in SNX1- and SNX2-depleted HeLa cells, despite efficient SNX knockdown (Figure 5c). Consistent with this result, no decrease in the ci-MPR signal was observed by immunofluorescence in depleted cells (Figure 5d). Nonetheless, a redistribution of the retromer component Vps26 (Figure 5d) that tightly associates with Vps35^42^ was observed, demonstrating that key retromer localization is altered by reducing SNX protein levels.

### SNX2 reduces Met phosphorylation and signaling

**Figure 6:**
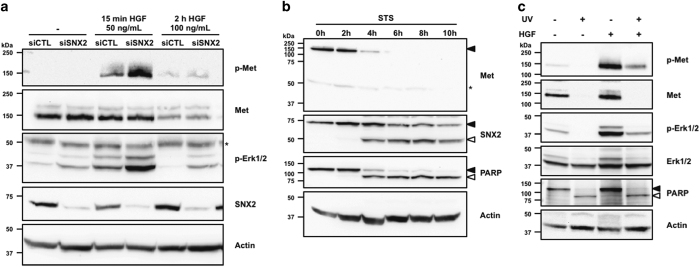
SNX2 depletion enhances Met signaling. (**a**) HeLa cells were transfected two times at a 24 h interval with control (siCTL) or a SNX2 small interfering RNA (siRNA). At 56 h, cells were serum-starved for 16 h and then stimulated with HGF as indicated. (**b**) HeLa cells were treated with STS (0.5 *μ*M) for the indicated period of time. (**c**) Serum-starved HeLa cells were treated with UV (100 J/m^2^, 16 h) and HGF (100 ng/ml, 15 min). Proteins were analyzed by immunoblotting using the indicated antibodies. Closed and open arrowheads indicate full-length and cleaved fragments, respectively. Actin was used as a loading control. Asterisks indicate nonspecific proteins recognized by the antibodies.

Another important function ascribed to SNX2 is in the trafficking of receptors, especially that of Met/HGFR.^34^ To assess the role of caspase-mediated cleavage of SNX2 on Met signaling, HeLa cells, which express endogenous Met, were transfected with SNX2-specific siRNA, and then treated with HGF (Figure 6a). Cells depleted in SNX2 showed more robust Met tyrosine phosphorylation as well as stronger Erk1/2 activation after 15 min of stimulation, but steady level of Met protein. However, stimulating cells for 2 h using a higher concentration of HGF resulted in the downregulation of Met that was not altered by SNX2 depletion, although some remnant Erk1/2 activation was detected.

Because Met is cleaved by caspases in apoptosis,^43,44^ the kinetics of Met and SNX2 cleavage were analyzed (Figure 6b). As predicted, Met was rapidly cleaved as full-length levels started to decrease after 4 h, and this decrease correlated well with that of SNX2. Finally, the combined effect of Met stimulation and apoptosis induction using UV on Met signaling was assessed (Figure 6c). Such treatment resulted in receptor disappearance and almost complete abrogation of Met signaling. These results suggest a potential detrimental role of SNX2 in diseases involving RTK signaling.

### SNX2 expression in CRC

**Figure 7:**
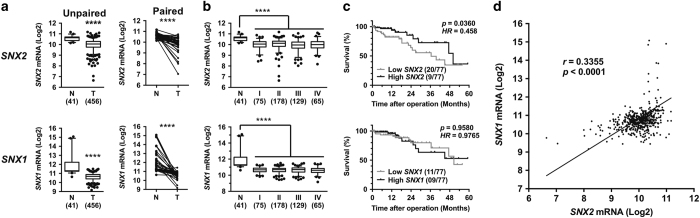
*SNX2* gene expression is decreased in CRC tumors. (**a**) *SNX2* and *SNX1* mRNA levels (unpaired, *l*eft panels; paired, right panels) in 41 normal (N) and 456 CRC tumor (T) samples from patients. Data were analyzed using Mann–Whitney (unpaired) or Wilcoxon's (paired) nonparametric test. (**b**) *SNX2* and *SNX1* mRNA levels categorized according to tumor stage. Data were analyzed using Kruskal–Wallis multiple comparison tests. Although no tumor stage shows significant difference with the others, all stages are significantly different from normal samples (*P*<0.0001). (**c**) Kaplan–Meier analysis of high 25% and low 25% *SNX2* and *SNX1* mRNA expression levels correlated with patient survival. Data were analyzed using the Mantel–Cox (log-rank) test and *P*-values and calculated Mantel–Haenszel HRs are indicated (see text). (**d**) Correlation between *SNX2* and *SNX1* mRNA expression levels in 497 samples. The Spearman's coefficient *r* and *P*-value are indicated. ****P<0.0001. See Supplementary Figure 2 for further analyses.

**Figure 8:**
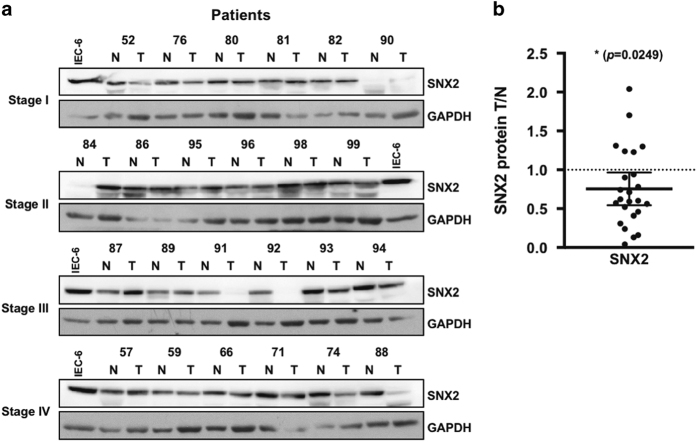
SNX2 protein levels are decreased in CRC tumors. (**a**) Samples from our CRC tissue bank were analyzed by immunoblotting for SNX2 protein in adjacent normal (N) and tumor (T) tissues. Glyceraldehyde 3-phosphate dehydrogenase (GAPDH) was used as a loading control to normalize data; the same IEC-6 (intestinal epithelial cell line) cell extract sample was used in all immunoblots to normalize results across different experiments. (**b**) Densitometric analysis of all CRC stages from (**a**). The *P*-value is indicated (*t*-test). *P<0.05.

The ability of SNX2 to reduce Met signaling suggests a possible role for this protein in cancer development. Interrogation of The Cancer Genome Atlas (TCGA) database^45^ revealed that *SNX2* mRNA expression is significantly decreased in CRC tumors compared with normal tissues; a similarly significant decrease in SNX1 mRNA was observed (Figure 7a). Both *SNX2* and *SNX1* mRNA levels were significantly decreased in stage I tumors and remained low at more advanced disease stages (Figure 7b and Supplementary Figure 2), suggesting an early and sustained alteration in CRC pathogenesis. The analysis of patient survival with the lowest quartile of *SNX2* expression shows a significant increase in mortality (Figure 7c). Indeed, the calculated hazard ratio (HR) indicates that these patients have approximately twice as much chance of dying than patients in the highest *SNX2* expression quartile. Such reduction in survival was not observed in patients with low levels of *SNX1* expression. Because both SNX1 and SNX2 have similar cellular roles and their expression levels are reduced in CRC tumors, their expression was analyzed for correlation and was significantly linked (Spearman's coefficient *r*=0.3355; Figure 7d). The exacerbation of Met signaling following SNX2 reduction suggests a potential role in CRC tumor development. Therefore, tumor samples from 24 patients of all CRC stages were analyzed for SNX2 protein and compared with adjacent normal tissues (Figure 8). Aggregation of all tumor stages shows a significant reduction in SNX2 protein.

## Discussion

Caspases recognize a sequence of four residues C terminus from the scissile bond, identified as P4 to P1, where P1 position corresponds to an aspartate residue in most substrates^5,46^ and the residue in P4 provides some specificity among caspases. Moreover, the position following P1, P1', is also important and is preferentially occupied by a small residue.^47^ In our study, we demonstrate that SNX2 proteolysis occurs at VSLD_84_↓S and we only observed a single fragment of 50 kDa, suggesting that SNX2 is cleaved at a single location. *In vitro* cleavage assays showed that initiator caspases 8–10, as well as executioner caspase-6, can perform the cleavage, which is consistent with the previously established substrate preferences.^46,47^ Furthermore, Asp_84_ is situated in the NTD of SNX2 that is unstructured, which is usually a requirement for caspase cleavage.

Using interfering RNA and ectopic expression techniques, we showed that caspase-6 contributes to SNX2 proteolysis by the extrinsic and intrinsic apoptotic pathways. Although the cleavage of the specific caspase-6 substrates was nearly completely blocked, significant SNX2 cleavage fragment remained, supporting a contribution by initiator caspases. However, overexpression experiments using XIAP or Bcl_XL_ to prevent caspase-9 activation following death receptor ligation and to isolate the contribution of initiator caspases 8 and 10 were inconclusive (data not shown). Caspase-6 is unique as its cleavage preference diverges from that of the other executioners and possesses fewer known substrates. During apoptosis, nuclear lamins, lamin B_1_ receptor, RIP kinase 1, CBP/p300 and vimentin are efficaciously cleaved by caspase-6, but not by the other caspases.^41,48–52^ Although it is not clear why cells have evolved an executioner caspase dedicated to a limited number of substrates, it is tempting to speculate that it is a way to ensure efficacy and timely proteolysis of key substrates by limiting the substrate repertoire to fewer candidates.

Contrary to SNX2, we demonstrated that SNX1 is cleaved at multiple sites *in vitro* by initiator caspases 8–10, but not by the executioners. Of the many fragments observed, two of them were detected in apoptotic cells. In addition to the previously reported site, we identified 15 new cleavage sites, 6 of which were following a glutamate residue. Cleavage at Asp_91_ (analogous to Asp_84_ in SNX2) is predicted to generate a 50-kDa fragment, which we detected both *in vitro* and *in cellulo*. Based on the identified sites, the 25-kDa fragment we observed in apoptotic cells most likely corresponds to proteolysis at LFAD_91_↓A and near the C terminus of the PX domain (MLQD_258_↓P, QDPD_260_↓V or DVRE_263_↓F). However, based on the current literature, cleavage at QDPD_260_↓V is the most likely site. By analogy to the cleavage of SNX2, the cleavage sites we identified in SNX1 better match the reported signature of initiator caspases and executioner caspase-6, although we did not observe proteolysis by the latter *in vitro*. Because of the location of many cleavage sites, we can assume that the effect on SNX1 function following its cleavage is similar to that of SNX2.

SNX2 associates with the retromer, a protein complex involved in endosome-to-TGN shuttling.^19,20^ In our study, we show that SNX2 lacking the first 84 residues mimicking the 50-kDa caspase-cleaved fragment failed to interact with Vps35 and that the N-terminal end no longer attaches to endosomal membranes, demonstrating that the NTD of SNX2 is responsible for its association with Vps35, the core component of the cargo-selective subcomplex. Moreover, SNX1 NTD alone associates to Vps35.^31^ Therefore, cleavage of SNX2 and by extension that of SNX1 decouples the retromer from the SNX-BAR subcomplex.

In HeLa cells, it was shown that SNX1 and SNX2 are required for the recruitment of the cargo-selective complex to the endosomal membrane and the retrieval of ci-MPR to the TGN.^20^ In that same study, depletion of both SNXs induced the redistribution of the Vps26 subunit from the endosomes to the cytosol and the lysosomal degradation of ci-MPR. Whereas we observed the redistribution of Vps26, we failed to detect such decrease in ci-MPR upon SNX1 and SNX2 depletion. In another study, depletion of SNX1 alone in HeLa cells was sufficient to affect the distribution of ci-MPR,^17^ a phenomenon that was not observed by initial studies.^20^ In other work, neither ci-MPR redistribution nor its cellular decrease was observed in tissues derived from *Snx1*- and *Snx2*-null mice.^21^ Finally, a study showed mislocalization and hasten degradation of ci-MPR in SNX1-depleted HeLa cells, as well as a reduced activity of lysosomal enzymes that are transported by MPRs.^22^ On the contrary, we failed to detect a decrease in cathepsin D in depleted cells (data not shown).

The dismantling of the cell during apoptosis requires the cleavage of many proteins to stop processes that are no longer required, potentially detrimental to the cell or work against cell death. Survival signals emanating from RTKs counteract cell death signals in many ways, including the direct phosphorylation of caspases by downstream kinases.^53^ Although with rare exceptions, phosphorylation usually inhibits or prevents normal activation of caspases.^53^ Thus, it is not surprising that dysregulation of RTK signaling occurs in many cancers. In our study, we show that SNX2 depletion followed by Met stimulation with HGF results in increased phosphorylation of both Met and downstream Erk1/2. In the context of cancer, Met activation induces diverse signaling cascades leading to cell proliferation, invasion, migration, angiogenesis and survival.^54,55^ Consequently, it is reasonable that the cleavage of SNX2 is accompanied by the inactivation of Met. Indeed, we showed that the onset of SNX2 cleavage correlates with the disappearance of full-length Met, which agrees with works by different groups.^44,56,57^

The modulation of RTKs by SNXs is not unique to HeLa cells. Indeed, SNX1 depletion in a non-small-cell lung cancer cells resistant to the EGFR inhibitor gefitinib showed a similar effect on Met and Erk1/2 phosphorylation.^33^ In similar cells overexpressing constitutively active Met, SNX2 depletion promotes the endocytosis of the receptor and its degradation.^34^ These conclusions seem also valid for other RTKs as work by Nguyen *et al*.^58^ showed an increase in EGFR phosphorylation following SNX1 depletion in CRC cells. That same group and others also observed a significant decrease of SNX1 protein in human CRC tumors,^58,59^ reinforcing the close link between SNXs and cancer.

Our findings raise the question of why caspases target SNX proteins during apoptosis in the first place. To reconcile the apparent contradiction between SNX role in healthy cells and the effect their cleavage has in apoptosis, we propose that we cannot consider their cleavage alone. For instance, concomitant cleavage of SNX2 and Met may tilt the balance towards apoptosis. However, it is also plausible that SNX2 cleavage has a crucial role under different conditions. As such, cleavage of proteins involved in intracellular trafficking is probably an efficacious mean to prevent the replication and spread of pathogens as studies have shown that SNX1 and SNX2 participate in these processes.^60,61^ On the other hand, it is not surprising that dysfunction of caspases such as caspase-6 and SNX/retromer components has been linked to a growing number of neurological disorders.^62,63^ Therefore, the identification and validation of the list of proteins that are altered by the caspase-mediated cleavage of specific death substrates will provide a fuller picture of the effect these cleavage events have as a part of a regulation mechanism or as a part of their dysfunction leading to pathogenesis.

## Materials and Methods

### Antibodies, reagents and chemicals

Monoclonal antibodies (mAbs) anti-PARP C2-10 (556362), anti-HSP90 68 (610418), anti-SNX1 51 (611482) and anti-SNX2 13 (611308) were purchased from BD Biosciences (San Jose, CA, USA). Anti-actin AC-40 (A4700) and anti-FLAG M2 (F3165) mAbs were from Sigma-Aldrich (Saint-Louis, MO, USA). Anti-SNX1 6H1 (H00006642-M01) mAb and polyclonal antibody (pAb) against SNX2 (PAB23106) were from Abnova (Taipei, Taiwan). Anti-HA 16B12 (MMS-101P) mAb was from Covance (Princeton, NJ, USA). Anti-lamin A and C JOL2 (ab40567) and anti-ci-MPR (ab124767) mAb, pAbs against SNX1 (ab995), Vps26 (ab23892) and RFP (ab62341) were from Abcam (Cambridge, UK). Anti-caspase-6 (no. 9762) and p-Erk1/2 Thr202/Tyr204 (no. 9101) pAbs, anti-Erk1/2 37A (no. 9107), anti-p-Met Y1234/1235 D26 (no. 3077) and anti-Myc 9B11 (no. 2276) mAbs were from Cell Signaling Technology (Danvers, MA, USA). Anti-Vps29 and anti-Vps35 pAbs were kind gifts from J Bonifacino. pAb anti-Met C-12 (sc-10) was from Santa Cruz Biotechnology (Dallas, TX, USA). mAb anti-mCherry (MO22140) was from Neuromics (Minneapolis, MN, USA). mAb anti-ci-MPR (MA1-066) and pAb anti-EEA1 (PA1-063A) were from Thermo Fisher Scientific (Waltham, MA, USA). Anti-Myc pAb (06-549) was from EMD Millipore (Billerica, MA, USA). Recombinant human-soluble TRAIL, STS, the irreversible caspase inhibitor Z-VAD-fmk and the irreversible thrombin inhibitor D-FPR-cmk were purchased from Enzo Life Sciences (Farmingdale, NY, USA). Recombinant human-soluble TNF-*α* was from Alexis Biochemicals (San Diego, CA, USA). Cycloheximide (CHX) and general protease inhibitors were from Sigma-Aldrich. Human HGF was from PeproTech (Rocky Hill, NJ, USA). General chemicals were from Sigma-Aldrich and Thermo Fisher Scientific.

### DNA constructs

SNX1 and SNX2 cDNAs were purchased from Origene Technologies (Rockville, MD, USA). These sequences were subcloned in a modified pGEX-KG vector containing a thrombin cleavage site to generate N-terminal GST-tagged SNX1 or SNX2 for purification. SNX2 cDNA was subcloned in pcDNA3 to generate pcDNA3-SNX2 WT and pcDNA3-SNX2Δ84. siRNA resistance was incorporated into SNX2 cDNA using the oligonucleotide 5′- CAGAGCTGCCCAGGGCAGTCAATACACAGGCTCTGAGTGG-3′ (mutated nucleotides are underlined). The Cherry fluorescent protein cDNA from the prSETBmCherry plasmid was subcloned into pcDNA3-SNX2 WT and Δ84 to generate pcDNA3-mCherry-SNX2 WT and Δ84. SNX2 residues 1–84 were amplified by PCR from pcDNA3-SNX2 WT plasmid and subcloned into the pEGFP-C1 plasmid. EGFP was replaced by subcloning Cherry from the prSETBmCherry plasmid to generate pmCherry-SNX2(1–84). Asp_84_ in SNX2 was mutated to an alanine residue to generate the cleavage-resistant mutant SNX2. The cDNA encoding residues 24–293 of caspase-6 were subcloned into pcDNA3-FLAG to generate pcDNA3-ΔN Casp6wt-FLAG (lacking the N-terminal peptide^64^), which was then used to create pcDNA3-ΔNCasp6-C285A-FLAG catalytic mutant by site-directed mutagenesis. The plasmid pCI-neo-Myc-Vps35 was a kind gift from J Bonifacino. The integrity of all constructs was confirmed by DNA sequencing.

### Cell culture, transfection and treatments

HeLa cells were purchased from ATCC and HEK293T cells were provided by A Newton (University of California, San Diego, CA, USA). Cells were maintained in Dulbecco’s modification Eagle’s medium supplemented with 10% fetal bovine serum, 2 mM l-glutamine and penicillin/streptomycin antibiotics (Wisent Inc., Saint-Bruno, QC, Canada). Lipofectamine 2000 (Thermo Fisher Scientific) and Fugene 6 (Promega, Fitchburg, WI, USA) were used for siRNA and plasmid DNA transfection, respectively, according to the manufacturer’s instructions. To induce apoptosis, cells were treated for the indicated period of time in fresh media containing the reagents indicated in the figure’s legends. UV radiation exposure was carried out in a Spectrolinker XL-1000 crosslinker with a minimal volume of media covering the cells during irradiation; fresh media were then added. For Met receptor stimulation, cells were serum starved for 16 h before HGF stimulation. For RNA interference and rescue, HeLa cells were transfected with a final concentration of 100 nM siRNA duplex according to the manufacturer’s instructions. Caspase-6 siRNAs (Dharmacon, Lafayette, CO, USA) were transfected and cells were used at 48 h or split and then used 24 h later depending on cell monolayer confluency. SNX1 and SNX2 siRNAs (Qiagen, Hilden, Germany) were transfected at 0 and 24 h, and cells were used at 72 h. For SNX2 rescue experiments, cells were transfected with plasmid DNA 10 h after the second siRNA transfection.

### Immunoblotting

Cells were lysed for an hour at 4 °C in RIPA buffer (50 mM Tris, pH 7.4, 100 mM NaCl, 1% NP-40, 0.5% deoxycholic acid, 0.1% SDS and 1 mM EDTA) with protease inhibitors (1 mM 1,10-orthophenantroline, 50 *μ*M 3,4-dichloroisocoumarine (DCI), 10 *μ*M E64 and 10 *μ*M leupeptin). For Met receptor experiments, cells were lysed using Triton lysis buffer (50 mM Tris, pH 7.5, 150 mM NaCl, 1% Triton X-100, 0.5% (w/v) deoxycholic acid, 1 mM phenylmethylsulfonyl fluoride (PMSF) and 1 mM sodium orthovanadate). Lysates were clarified at 18 000×*g* for 15 min and protein concentration was determined using Pierce BCA Protein Assay Kit (Thermo Fisher Scientific). Protein samples were separated on 12% ammediol SDS-PAGE^65^ and transferred to nitrocellulose (Perkin-Elmer, Waltham, MA, USA) or PVDF membranes (EMD Millipore).^66^ Membranes were blocked in PBS containing 0.1% Tween-20 and 5% non-fat dry milk or 5% BSA. Ensuing membranes were incubated with primary antibodies and horseradish peroxidase-conjugated goat anti-rabbit or horse anti-mouse IgG secondary antibodies (Cell Signaling Technology). Membranes were revealed by chemiluminescence using Luminata Crescendo (EMD Millipore).

### Microscopy

HeLa cells grown on coverslips for 18 h were fixed for 30 min with 3% paraformaldehyde in 100 mM phosphate buffer (pH 7.4), permeabilized in 0.1% Triton X-100 for 10 min and blocked with 10% goat serum for 30 min. Cells were then sequentially incubated for an hour at room temperature with primary antibodies and Alexa Fluor-conjugated secondary antibodies (Molecular Probes, Eugene, OR, USA). An inverted confocal laser scanning microscope (FV1000; Olympus, Tokyo, Japan) equipped with a PlanApo x60/1.42 oil immersion objective (Olympus) was used to visualize the samples and Olympus FluoView software version 1.6a was employed for image acquisition and analysis. Images were processed with Adobe Photoshop (Adobe Systems, San Jose, CA, USA).

### Immunoprecipitation

HEK293T cells were transfected with plasmid DNA for 48 h. Cells were lysed in RIPA buffer and incubated 18 h with primary antibodies at 4 °C. Cell lysates were then incubated with protein A-sepharose (GE Healthcare, Marlborough, MA, USA) for an hour at 4 °C and beads were washed three times with lysis buffer. Bound immune complexes were boiled in SDS-PAGE loading buffer.

### Recombinant protein expression and purification

GST-tagged SNX1 and SNX2 were expressed in BL21(DE3) *pLysS Escherichia coli* using 0.1 mM IPTG for 18 h at 18 °C. Bacteria were recovered by centrifugation and stored overnight at −80 °C in PBS containing 1 mM PMSF. Cell lysis was carried out by sonication in PBS plus protease inhibitors (2 mM PMSF, 2 mM EDTA, 100 *μ*M tosyl-l-lysyl-chloromethane hydrochloride, 10 *μ*M 3,4-DCI and 10 *μ*M E64) and then incubated with 1% Triton X-100 for 45 min. The lysate was centrifuged for 20 min at 18 000×*g* and the supernatant was incubated with glutathione sepharose 4B beads (GE Healthcare) for an hour at 4 °C. Beads were washed four times with PBS containing 1% Triton X-100 and treated for 18 h with thrombin (T-6634; Sigma-Aldrich) in PBS with 0.2% Triton X-100 to cleave the GST moiety. Thrombin was inhibited with 1 *μ*M D-FPR-cmk and proteins were stored at −80 °C. Recombinant caspases were expressed, purified and active site-titrated as described elsewhere.^67^

### SNX1 and SNX2 cleavage by caspases

Caspase cleavage assays were performed for 30–60 min at 37 °C at a final SNX1 or SNX2 concentration of 25 nM in either executioner caspase buffer (10 mM PIPES, pH 7.2, 100 mM NaCl, 10 mM DTT, 1 mM EDTA, 10% sucrose and 0.1% CHAPS) or in high salt initiator caspase buffer (50 mM HEPES, pH 7.4, 1 M sodium citrate, 50 mM NaCl, 10 mM DTT and 0.01% Chaps).^68^ Initiator caspases were preincubated for an hour (2 h for caspase-2) in high salt buffer before performing the assays. Enzymatic reactions were stopped by adding SDS-PAGE loading buffer solution and boiling (executioner caspases) or by TCA precipitation to remove excess salt (initiator caspases).

### Mass spectrometry

Five hundred nanomoles of recombinant SNX1 were incubated with or without 100 nM of recombinant caspase-10 for an hour at 37 °C. TCA-precipitated samples were made 1 mg/ml in 10 mM HEPES, 8 M urea and 1 *μ*g DTT. Samples were boiled at 95 °C for 2 min and further incubated at room temperature for 30 min. Five micrograms of iodoacetamide was added and incubated at room temperature for 20 min. Samples were diluted to reduce urea concentration to 2 M using 50 mM NH_4_NCO_3_ and incubated with 1 *μ*g trypsin (Trypsin Gold V5280; Promega) at 30 °C overnight. Samples were acidified by adding TFA to 0.1% and purified using ZipTips. Samples were analyzed by tandem mass spectrometry as described previously.^69^ Algorithms were set to recognized peptides ending in Lys/Arg/Asp and Lys/Arg/Asp/Glu or following these residues with up to eight missed cleavage sites.

### CRC samples

The CRC sample biobank used is described elsewhere.^70^ Briefly, tissue samples were obtained following patient's written informed consent according to a protocol approved by the Institutional Human Subject Review Board of the Centre Hospitalier Universitaire de Sherbrooke (CHUS; protocol 07-089). Tissue extracts were prepared as described previously.^71^ Densitometric analyses were performed using a combination of the NIH Image J software and QuantiOne imaging system from Bio-Rad (Hercules, CA, USA). All immunoblotting data were normalized first using the GAPDH signal on the same membrane and then using the same IEC-6 sample loaded on every experiment to establish tumor/normal tissue ratios for each patient.

### Statistical analyses

Tumor mRNA expression data and linked clinical data sets were from TCGA,^45^ a publically available HiSeq RNA sequencing gene expression profiling data sets of human CRC samples. All data sets were first tested for normality using D’Agostino–Pearson and Wilk–Shapiro normality tests. Only when both normality tests were positive for all data sets to be compared did we use parametric tests. Otherwise, nonparametric tests were used. The test applied to data sets are indicated in figure legends. Statistical analyses were carried out using the GraphPad Prism v.7 software. Asterisks indicate statistical significance with *P*-values: *P⩽0.05; **P⩽0.01; ***P⩽0.001; and ****P⩽0.0001.
