# Supplementary material for: Caspase-mediated proteolysis of the sorting nexin 2 disrupts retromer assembly and potentiates Met/hepatocyte growth factor receptor signaling
Source: Cell Death Discov. 2017 Jan 23;3:16100–. doi: 10.1038/cddiscovery.2016.100 (PMC5253419; doi:10.1038/cddiscovery.2016.100)
Supplement: Supplementary Information [file cddiscovery2016100-s1.pdf]

Supplementary Figure 1

A

Thrombin-cleaved GST-SNX1 used for MS

```
GSPMASGGGGCSASERLPPPFPGLEPESEGAAGGSEPEAGDSDTEGEDIFTGAAVVSKHQ
SPKITTSLLPINNGSKENGIHEEQDQEPQDLFADATVELSLDSTQNNQKKVLAKTLISLP
PQEATNSSKPQPTYEELEEEEQEDQFDLTVGITDPEKIGDGMNAYVAYKVTTQTSLPLFR
SKQFAVKRRFSDFLGLYEKLSEKHSQNGFIVPPPPEKSLIGMTKVKVGKEDSSSAEFLEK
RRAALERYLQRIVNHPTMLQDPDVREFLEKEELPRAVGTQTLSGAGLLKMFNKATDAVSK
MTIKMNESDIWFEEKLQEVECEEQRLRKLHAVVETLVNHRKELALNTAQFAKSLAMLGSS
EDNTALSRALSQLAEVEEKIEQLHQEQANNDFFLLAELLSDYIRLLAIVRAAFDQRMKTW
QRWQDAQATLQKKREAEARLLWANKPDKLQQAKDEILEWESRVTQYERDFERISTVVRKE
VIRFEKEKSKDFKNHVIKYLETLLYSQQQLAKYWEAFLPEAKAIS
```

B

#	Peptide sequence	SNX1 D/E ↓	cleavage window (-8,+8)	Missed cleavages	Intensity SNX1 only	Intensity SNX1+casp-10
	**Cleavage following Asp**	**10**		D/K/R		
1	SDTEGEDIFTGAAVVSK	38	GSEPEAGD ↓ **SDTEGEDI**	2	0.00E+00	2.57E+09
2	LTVGITDPEK	144	EEQEDQFD ↓ **LTVGITDP**	1	0.00E+00	4.71E+08
3	TEGEDIFTGAAVVSK	40	EPEAGDSD ↓ **TEGEDIFT**	1	0.00E+00	1.91E+08
4	ATVELSLDSTQNNQKK	91	EPQDLFAD ↓ **ATVELSLD**	2	0.00E+00	1.90E+08
5	ATVELSLDSTQNNQK	91	EPQDLFAD ↓ **ATVELSLD**	1	0.00E+00	1.87E+08
6	TLISLPPQEATNSSKPQPTYEELEEEEQED	141	**LEEEEQED** ↓ QFDLTVGI	1	0.00E+00	1.70E+08
7	IFTGAAVVSK	45	DSDTEGED ↓ **IFTGAAVV**	0	0.00E+00	1.62E+08
8	ENGIHEEQDQEPQDLFAD	91	**EPQDLFAD** ↓ ATVELSLD	2	0.00E+00	8.99E+07
9	LPPPFPGLEPESEGAAGGSEPEAGD	38	**GSEPEAGD** ↓ SDTEGEDI	0	0.00E+00	5.58E+07
10	PEKIGDGMNAYVAYK	151	DLTVGITD ↓ **PEKIGDGM**	2	0.00E+00	3.58E+07
11	LTVGITDPEKIGDGMNAYVAYK	144	EEQEDQFD ↓ **LTVGITDP**	3	0.00E+00	3.53E+07
12	QFDLTVGITDPEK	141	LEEEEQED ↓ **QFDLTVGI**	2	0.00E+00	2.82E+07
13	VREFLEKEELPR	260	PTMLQDPD ↓ **VREFLEKE**	2	0.00E+00	2.75E+06
14	PDVREFLEKEELPR	258	NHPTMLQD ↓ **PDVREFLE**	3	0.00E+00	2.36E+06
15	KLQQAKDEILEWESR	444	LLWANKPD ↓ **KLQQAKDE**	3	0.00E+00	1.84E+06
	**Cleavage following Glu**	**6**		D/E/K/R		
16	AGDSDTEGEDIFTGAAVVSK	35	AAGGSEPE ↓ **AGDSDTEG**	5	0.00E+00	5.96E+08
17	SEGAAGGSEPEAGDSDTEGEDIFTGAAVVSK	24	PFPGLEPE ↓ **SEGAAGGS**	8	0.00E+00	4.41E+08
18	GEDIFTGAAVVSK	42	EAGDSDTE ↓ **GEDIFTGA**	2	0.00E+00	4.17E+07
19	LPPPFPGLEPE	24	**PFPGLEPE** ↓ SEGAAGGS	1	0.00E+00	4.06E+07
20	TLISLPPQEATNSSKPQPTYEELEEE	137	**TYEELEEE** ↓ EQEDQFDL	6	0.00E+00	1.79E+07
21	LPPPFPGLEPESE	26	**PGLEPESE** ↓ GAAGGSEP	2	0.00E+00	6.70E+06
22	FLEKEELPR	263	LQDPDVRE ↓ **FLEKEELP**	4	0.00E+00	2.56E+06

C

#1

Raw file	Scan	Method	Score	m/z	Gene names
242Catherine_Caspase10	57235	FTMS; HCD	97.9	863.41	SNX1

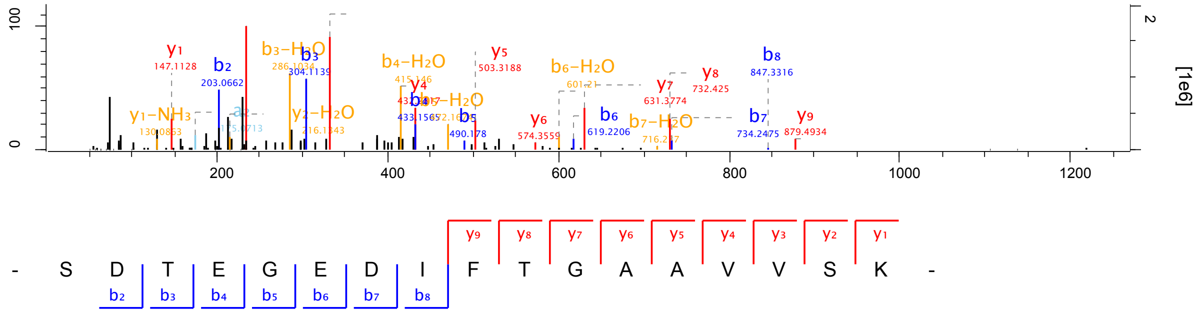

#2

Raw file	Scan	Method	Score	m/z	Gene names
242Catherine_Caspase10	35111	FTMS; HCD	108.47	536.8	SNX1

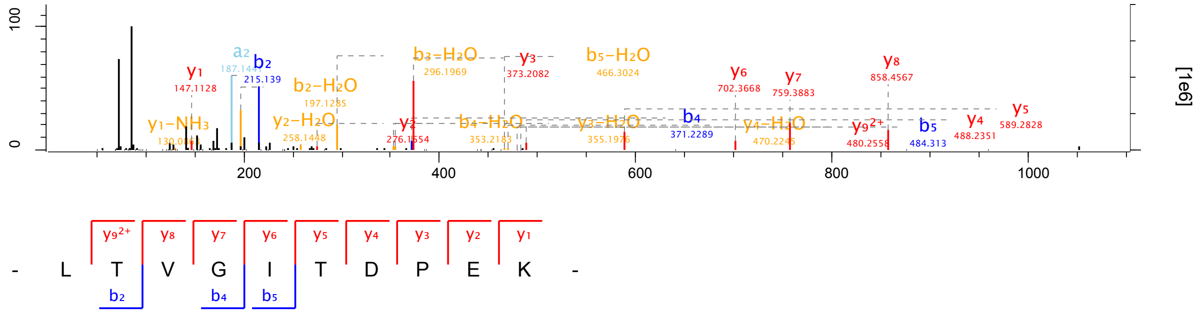

#3

Raw file	Scan	Method	Score	m/z	Gene names
242Catherine_Caspase10	54273	FTMS; HCD	59.71	762.38	SNX1

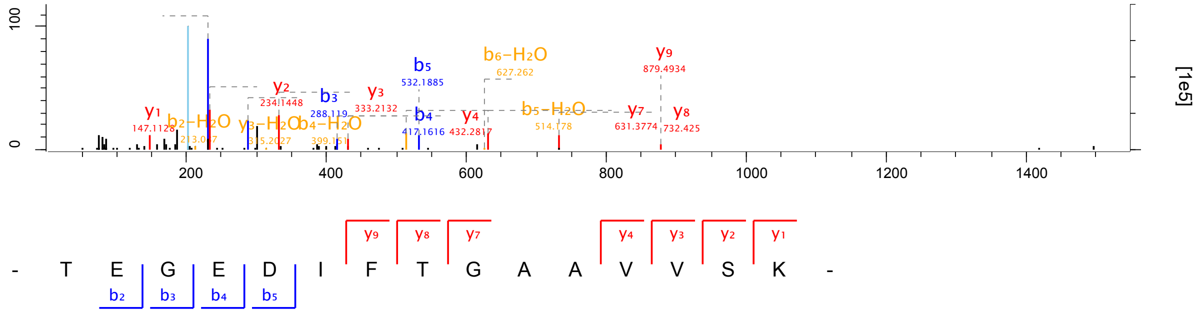

#4

Raw file	Scan	Method	Score	m/z	Gene names
242Catherine_Caspase10	25593	FTMS; HCD	123.29	592.64	SNX1

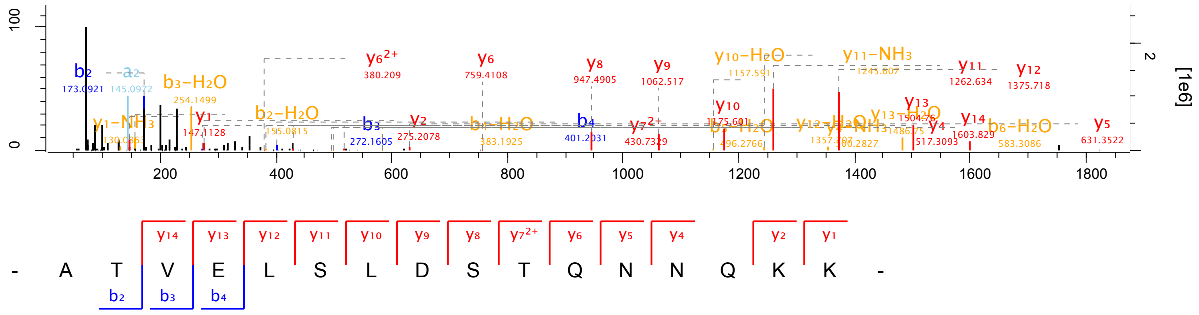

C (continued)

#5

Raw file	Scan	Method	Score	m/z	Gene names
242Catherine_Caspase10	36545	FTMS; HCD	98.34	824.41	SNX1

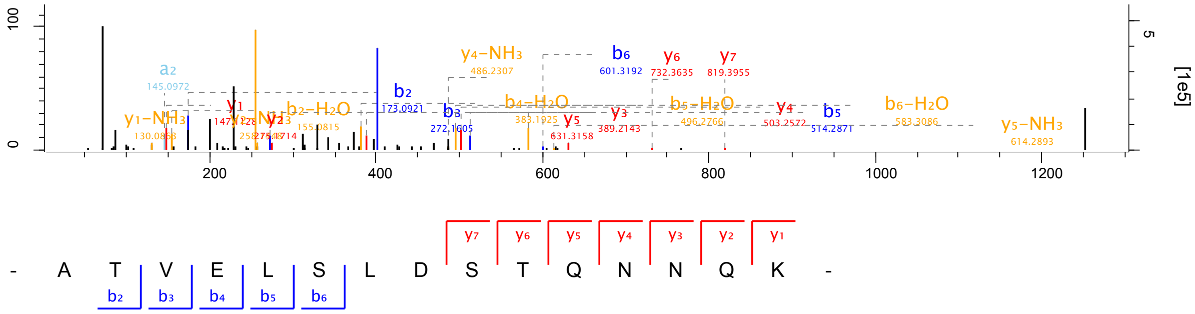

#6

Raw file	Scan	Method	Score	m/z	Gene names
242Catherine_Caspase10	70516	FTMS; HCD	17.86	1144.87	SNX1

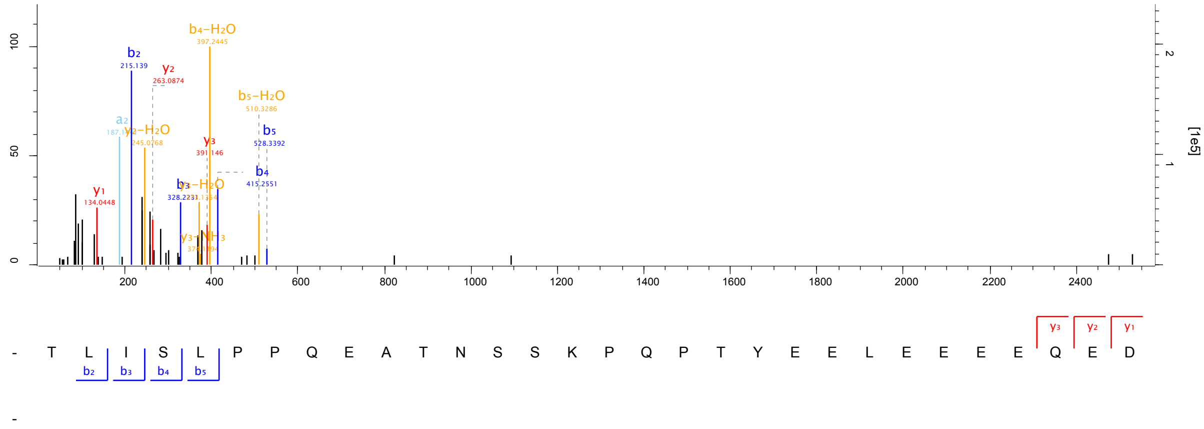

#7

Raw file	Scan	Method	Score	m/z	Gene names
242Catherine_Caspase10	30882	FTMS; HCD	79.66	496.79	SNX1

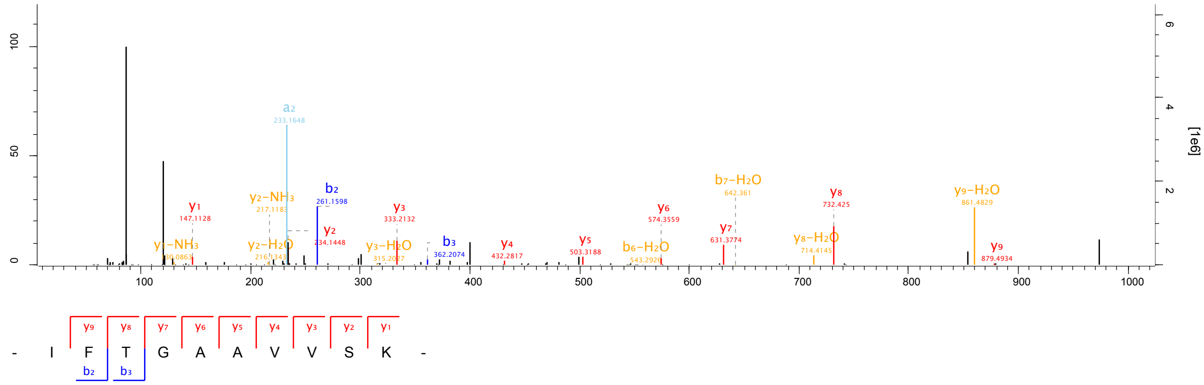

#8

Raw file	Scan	Method	Score	m/z	Gene names
242Catherine_Caspase10	54922	FTMS; HCD	16.31	705.64	SNX1

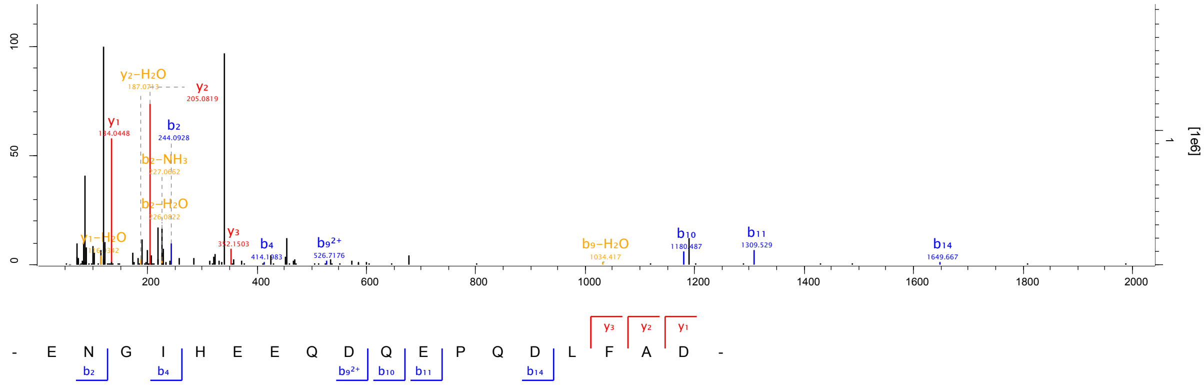

C (continued)

#9

Raw file	Scan	Method	Score	m/z	Gene names
242Catherine_Caspase10	83893	FTMS; HCD	19.4	803.37	SNX1

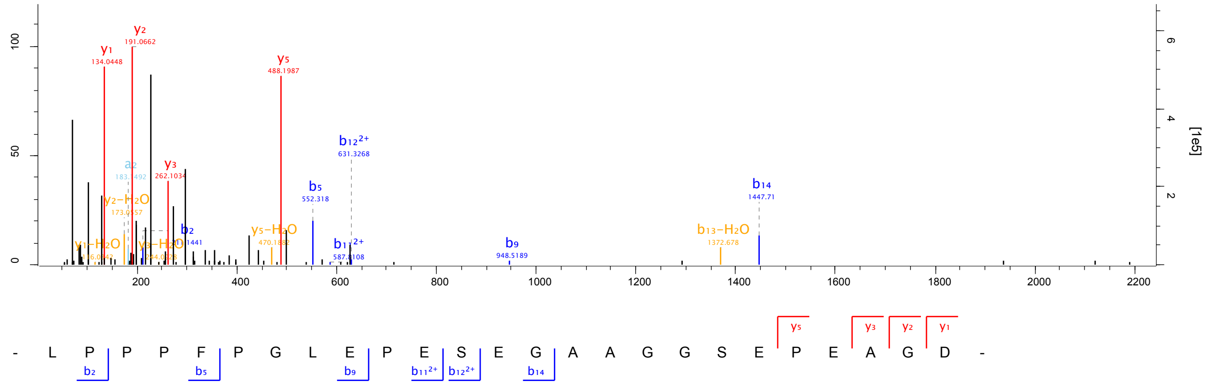

#10

Raw file	Scan	Method	Score	m/z	Gene names
242Catherine_Caspase10	38593	FTMS; HCD	85.01	552.94	SNX1

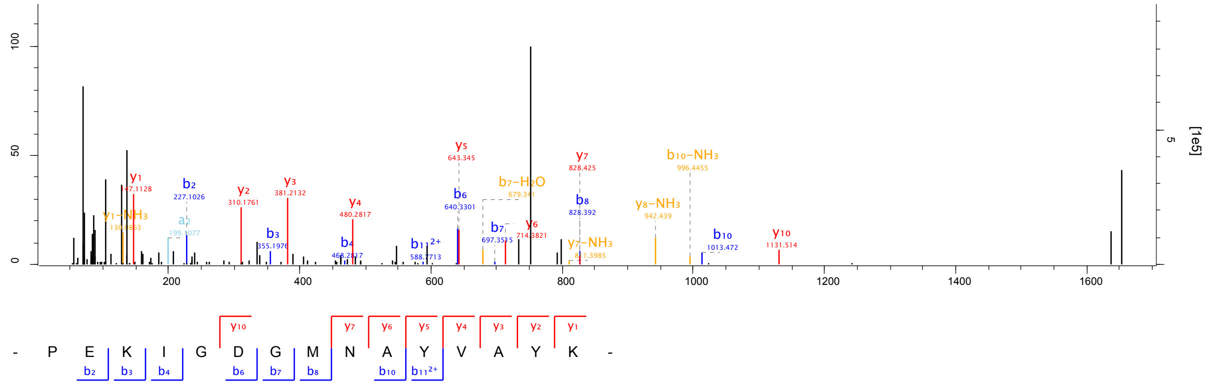

#11

Raw file	Scan	Method	Score	m/z	Gene names
242Catherine_Caspase10	68788	FTMS; HCD	18.32	786.07	SNX1

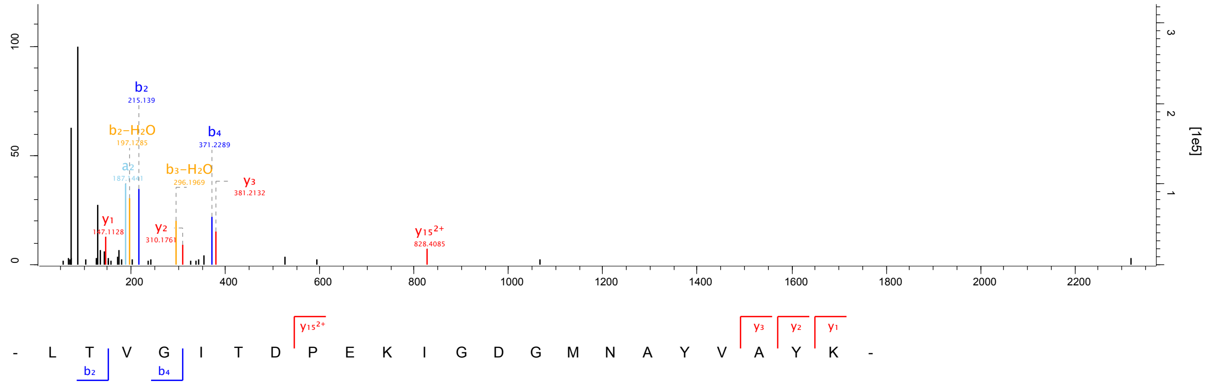

#12

Raw file	Scan	Method	Score	m/z	Gene names
242Catherine_Caspase10	64586	FTMS; HCD	68	731.38	SNX1

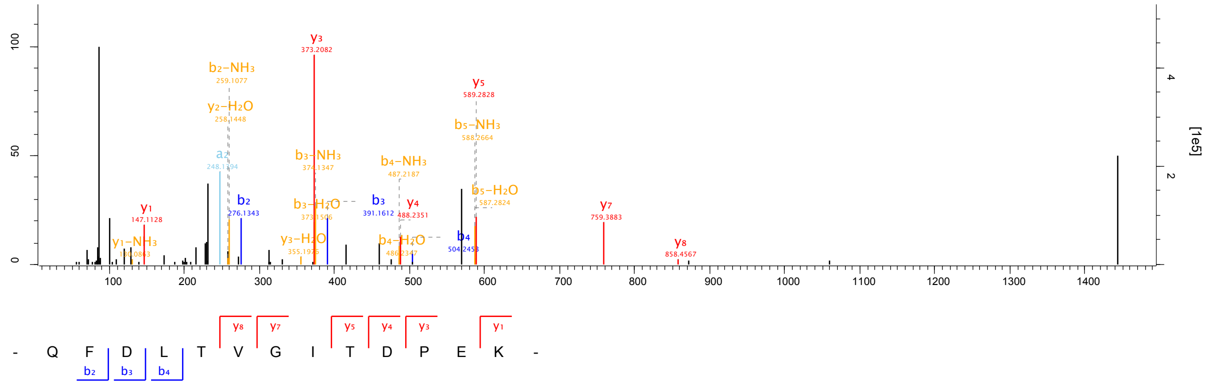

C (continued)

#13

Raw file	Scan	Method	Score	m/z	Gene names
242Catherine_Caspase10	30481	FTMS; HCD	25.98	387.72	SNX1

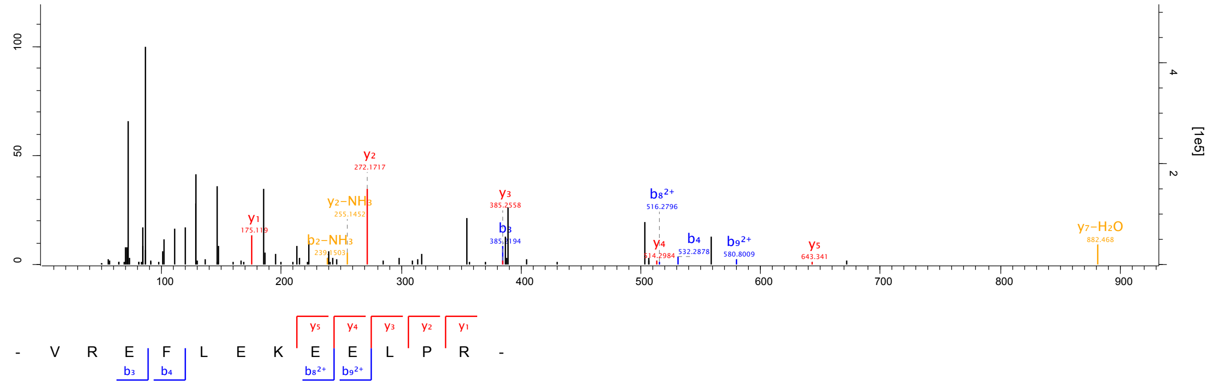

#14

Raw file	Scan	Method	Score	m/z	Gene names
242Catherine_Caspase10	55807	FTMS; HCD	18.82	586.98	SNX1

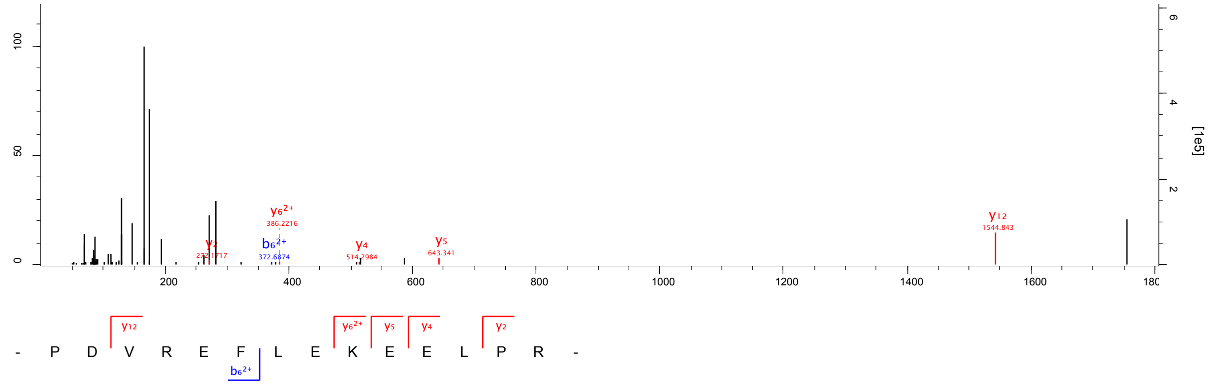

#15

Raw file	Scan	Method	Score	m/z	Gene names
242Catherine_Caspase10	43791	FTMS; HCD	12.2	469.25	SNX1

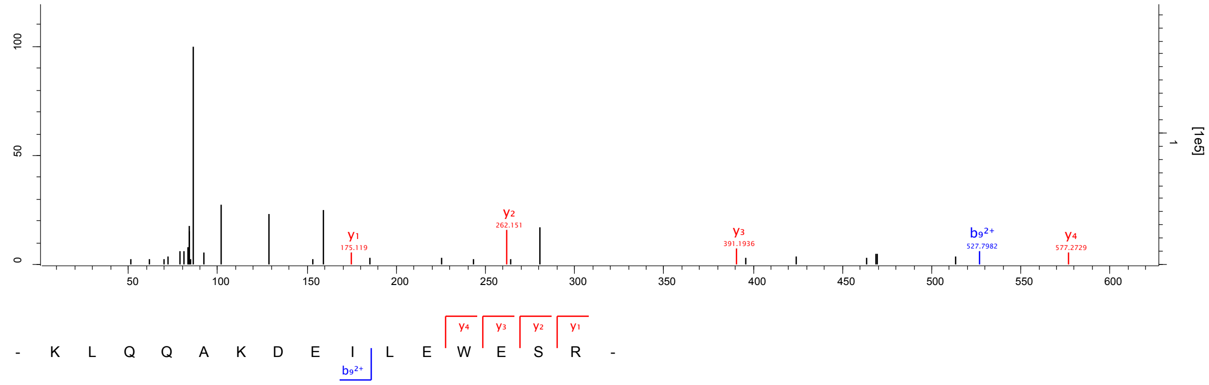

#16

Raw file	Scan	Method	Score	m/z	Gene names
242Catherine_Caspase10	58896	FTMS; HCD	65.02	657.31	SNX1

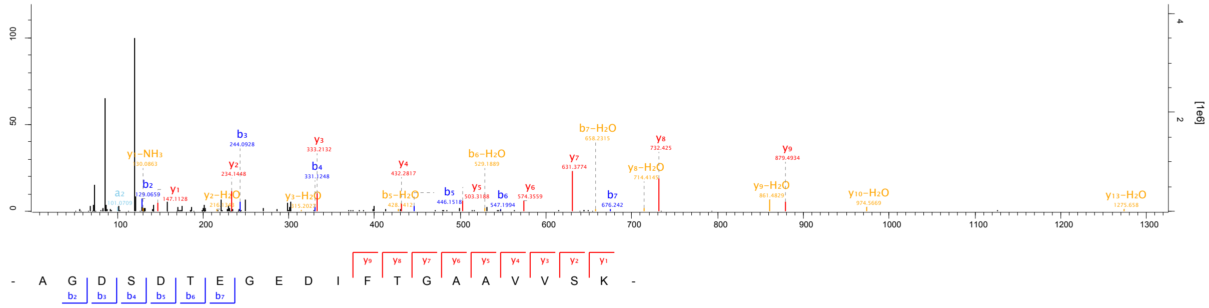

C (continued)

#17

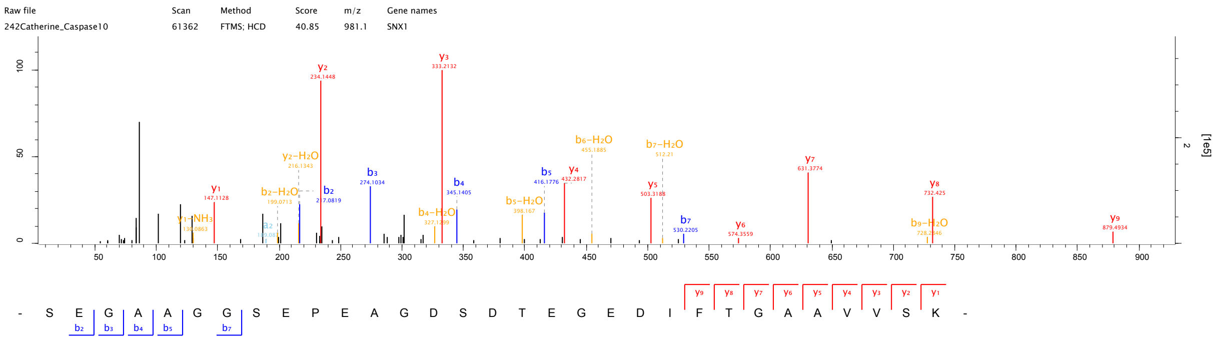

#18

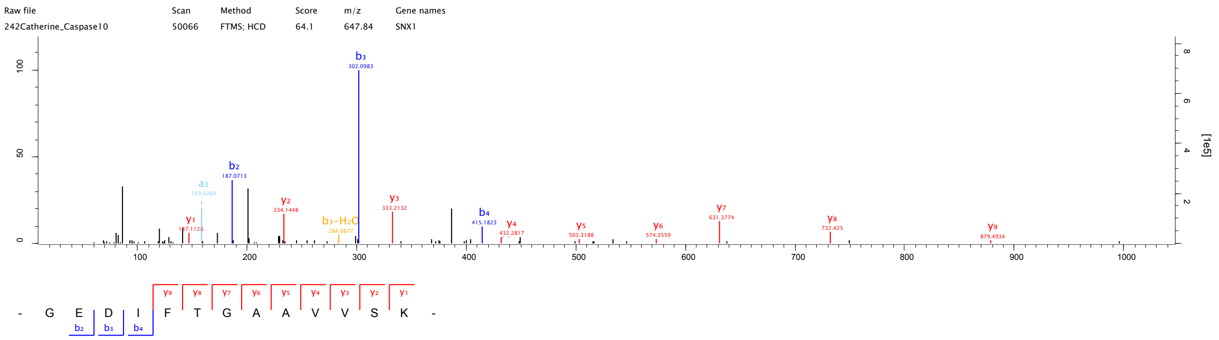

#19

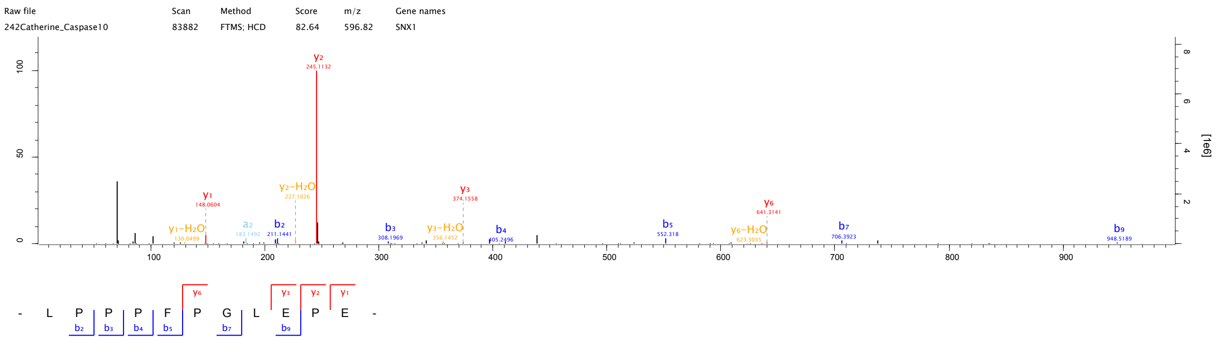

#20

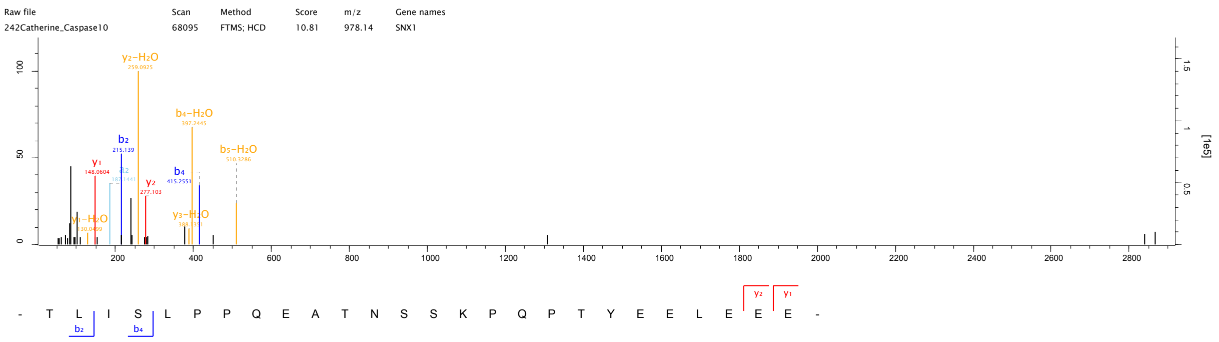

C (continued)

#21

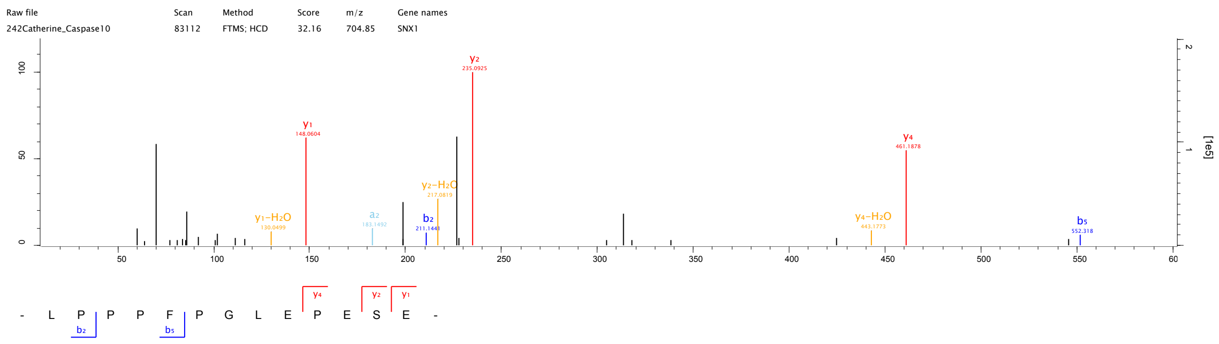

#22

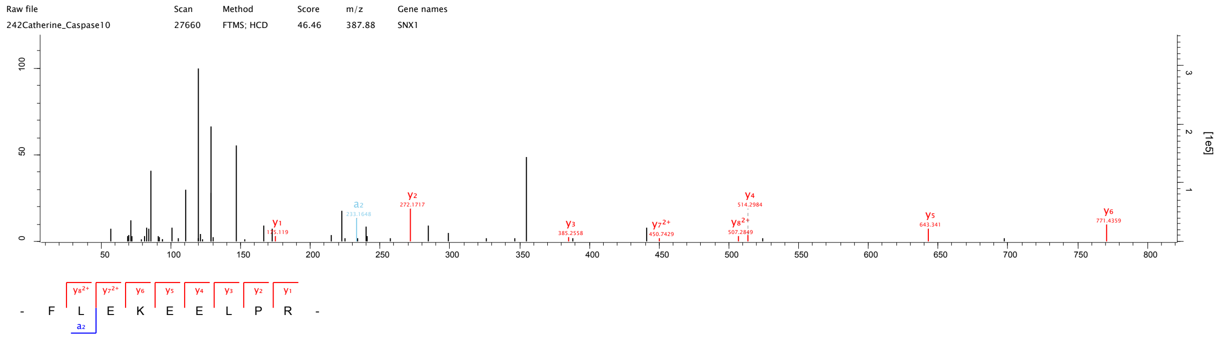

Supplementary Figure 1 Spectrometric analysis of SNX1 cleavage sites. (**a**) Primary structure of the human SNX1 protein used for the identification of cleavage sites. Peptide coverage (387/525, 73.4%) is shown in gray; aspartate and glutamate residues are in bold typeface; cleaved Asp and cleaved Glu sites are underlined and green and red typeface, respectively. The previously reported cleavage site is boxed and corresponds to Asp_{91}. The N-terminal GSP residues are from the fusion protein used to purify SNX1. (**b**) List of peptides identified that reveal cleavage sites. Cleaved aspartate/glutamate residues are identified (Uniprot: Q13596) along with [-8,+8] sequence window with cleavage site, missed Asp/Glu/Lys/Arg cleavages and peak intensities in the control and caspase-10-cleaved samples. Cleavage sites at $Asp_{38,91,141}$ and at Glu_{24} were identified by both N- and C-terminal peptides (brackets). (**c**) Peptide ion trap CID fragmentation for the peptides identified in **b**. Spectra are shown as matched to peptide sequences and annotated by MaxQuant 1.5.2.8.

Supplementary Figure 2

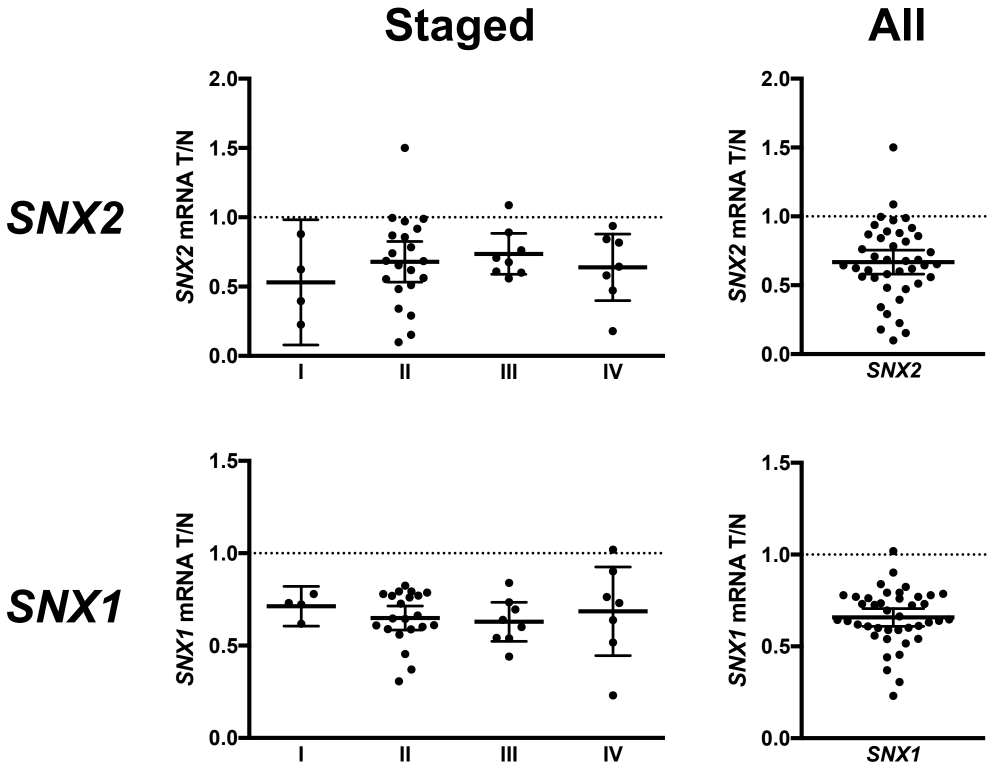

Supplementary Figure 2 *SNX2* gene expression is decreased in CRC tumors. *SNX2* and *SNX1* mRNA level tumor/normal ratios (staged, *left panel*; all stages, *right panel*) in CRC tumors from patients. Accompanies Figure 7.
